# Verification of a comprehensive framework for mobility using data from the Canadian Longitudinal Study on Aging: a structural equation modeling analysis

**DOI:** 10.1186/s12877-023-04566-x

**Published:** 2023-12-08

**Authors:** Sandra C. Webber, Yixiu Liu, Depeng Jiang, Jacquie Ripat, Scott Nowicki, Robert Tate, Ruth Barclay

**Affiliations:** 1 Department of Physical Therapy, College of Rehabilitation Sciences, Rady Faculty of Health Sciences, University of Manitoba, R106-771 McDermot Ave, Winnipeg, MB R3E 0T6 Canada; 2 Department of Community Health Sciences, Max Rady College of Medicine, Rady Faculty of Health Sciences, University of Manitoba, Winnipeg, Canada; 3 Department of Occupational Therapy, College of Rehabilitation Sciences, Rady Faculty of Health Sciences, University of Manitoba, Winnipeg, Canada

**Keywords:** Ambulation, CLSA, Life space, Transportation

## Abstract

**Background:**

Mobility within and between life spaces is fundamental for health and well-being. Our objective was to verify a comprehensive framework for mobility.

**Methods:**

This was a cross-sectional study. We used structural equation modeling to estimate associations between latent factors with data from the Canadian Longitudinal Study on Aging for participants 65–85 years of age (65+, n = 11,667) and for adults with osteoarthritis (OA) aged 45–85 (n = 5,560). Latent factors included life space mobility, and physical, psychosocial, environmental, financial, and cognitive elements. Personal variables (age, sex, education) were covariates.

**Results:**

The models demonstrated good fit (65+: CFI = 0.90, RMSEA (90% CI) = 0.025 (0.024, 0.026); OA: CFI = 0.90, RMSEA (90% CI) = 0.032 (0.031, 0.033)). In both models, better psychosocial and physical health, and being less afraid to walk after dark (observed environmental variable) were associated with greater life space mobility. Greater financial status was associated with better psychosocial and physical health. Higher education was related to better cognition and finances. Older age was associated with lower financial status, cognition, and physical health. Cognitive health was positively associated with greater mobility only in the 65 + model. Models generated were equivalent for males and females.

**Conclusions:**

Associations between determinants described in the mobility framework were verified with adults 65–85 years of age and in an OA group when all factors were considered together using SEM. These results have implications for clinicians and researchers in terms of important outcomes when assessing life space mobility; findings support interdisciplinary analyses that include evaluation of cognition, depression, anxiety, environmental factors, and community engagement, as well as physical and financial health. Public policies that influence older adults and their abilities to access communities beyond their homes need to reflect the complexity of factors that influence life space mobility at both individual and societal levels.

**Supplementary Information:**

The online version contains supplementary material available at 10.1186/s12877-023-04566-x.

## Background

Maintaining the ability to move is fundamental to health and social participation for older adults. Individuals with mobility limitations are at increased risk for illness and injury [1], being homebound or institutionalized [2], and experiencing reduced quality of life [3]. While the prevalence of mobility limitations differs depending on how mobility is defined and measured, mobility issues are common and represent significant problems for many older adults. 40% of older Americans report severe or moderate limitations related to difficulties with balance, walking, and climbing stairs [4] and 24% of Canadians aged 65 and older report limitations in daily activities such as moving around [5]. Older adults are at greater risk of functional limitation when they stop driving a vehicle [6, 7], and those who no longer drive often have difficulty continuing to be active in the community [8].

While mobility limitations are common in the general older population, physical impairments such as reduced strength and range of motion are prevalent in people with hip and knee osteoarthritis (OA), often resulting in specific functional limitations in walking [9]. People with OA are at increased risk for poor health [10], and higher levels of OA pain are associated with restrictions in life-space mobility [11].

It is important to understand factors that contribute to mobility limitations to improve health and well-being at individual and societal levels. Webber and colleagues introduced a theoretical framework to portray components that influence mobility across different life spaces expanding from the home to broader communities [12]. This framework suggests that mobility is influenced by financial, psychosocial, environmental, physical, cognitive, and personal factors.

Many studies have used the theoretical framework [12] to examine mobility in older adults. For example, a recent cross-sectional study in the United States demonstrated numerous variables representing key determinants in the framework were significantly associated with life space [13]. Personal, physical, and psychosocial factors explained 23.8% of the variance in self-reported life space attainment. Canadian Longitudinal Study on Aging (CLSA) analyses demonstrated that variables representing personal, physical, psychosocial, and cognitive domains were all correlated with life space [14]. Relationships have also been examined with objective mobility data collected with smartphones in older adults [15]. Most variables representing personal, psychological, physical, social, and cognitive domains were significantly correlated with one or more of the objective measures (e.g., walking time, steps, life-space area, total distance).

Despite widespread use of the theoretical mobility framework, only one study has examined its validity using structural equation modeling (SEM). Umstattd Meyer and colleagues (2014) used SEM to model personal mobility (physical domain) and community mobility (driving and availability of a vehicle) in 6,112 older Americans [16]. The final model demonstrated direct associations between personal, environmental, physical, and cognitive factors with mobility. Finances did not contribute substantially in the presence of other predictors, and psychosocial aspects exerted influence through relationships with cognition.

SEM is a powerful analytical tool that tests hypothetical relationships between theoretical constructs and between the constructs and their observed measures [17]. Because SEM considers multiple variables simultaneously and uses latent factors which reduce measurement error, it is superior to other correlation analyses such as regression [17]. Many researchers have demonstrated relationships between one or more specific personal, cognitive, physical, psychosocial, environmental and financial factors with life space mobility (for example [13, 14, 18, 19]). However, model verification using SEM allows for multiple indicator variables (self-reported and observed) and covariates to be examined together, and to determine relationships between indicator variables and latent factors. This provides information about direct and indirect associations with life space mobility and provides additional insight into relationships among latent factors. It is important to determine whether the theoretical framework can be applied to real world data, i.e., to see if relationships are as proposed. This type of evaluation can provide valuable information to clinicians (e.g., regarding relevant outcome measures and aspects to address in rehabilitation programs), and to researchers by providing further knowledge to frame and conceptualize life space research focusing on important contributing factors and impactful interventions. Community agencies that promote programs for older adults and policy makers also benefit from having a comprehensive understanding of the inter-play of factors that influence older adults’ abilities to engage in activities with differing mobility requirements.

In this study we were interested in verifying the theoretical mobility framework with data from the large CLSA data set [20–22]. Our objectives were to estimate associations between latent factors associated with life space mobility in older adults 65–85 years of age and in adults with OA (aged 45–85).

## Methods

The CLSA population-based longitudinal study includes two cohorts (Tracking and Comprehensive) aged 45–85 at baseline [20–22]. People excluded from participating included those unable to communicate in English or French, people living in long-term care and those with cognitive impairments, full-time members of the Canadian Forces, and individuals who resided in the three Canadian Territories, on Federal First Nation Reserves and in First Nation settlements [20–22]. The CLSA protocol was approved by 13 research ethics boards in Canada. All participants provided informed written consent. Ethics approval for this secondary analysis of the CLSA dataset was obtained from the Health Research Ethics Board at the University of Manitoba (HS22810 (H2019:173)).

### Study samples

Samples for this study were obtained from the 30,097 participants in the CLSA Comprehensive Group (Dataset version 4.0, collected 2011–2015). Data were obtained from the following sources: (1) In-Home Baseline Questionnaires; (2) Data Collection Site Questionnaires; (3) Physical Assessments at Data Collection Sites; and (4) Maintaining Contact Questionnaires (Wave 1 Version). We developed models for people 65–85 years of age (65+, n = 12,646 reduced to n = 11,667 when missing records were removed), and people with OA aged 45–85 (OA, n = 5,944 reduced to n = 5,560 when missing records were removed).

### Measures

**Fig. 1:**
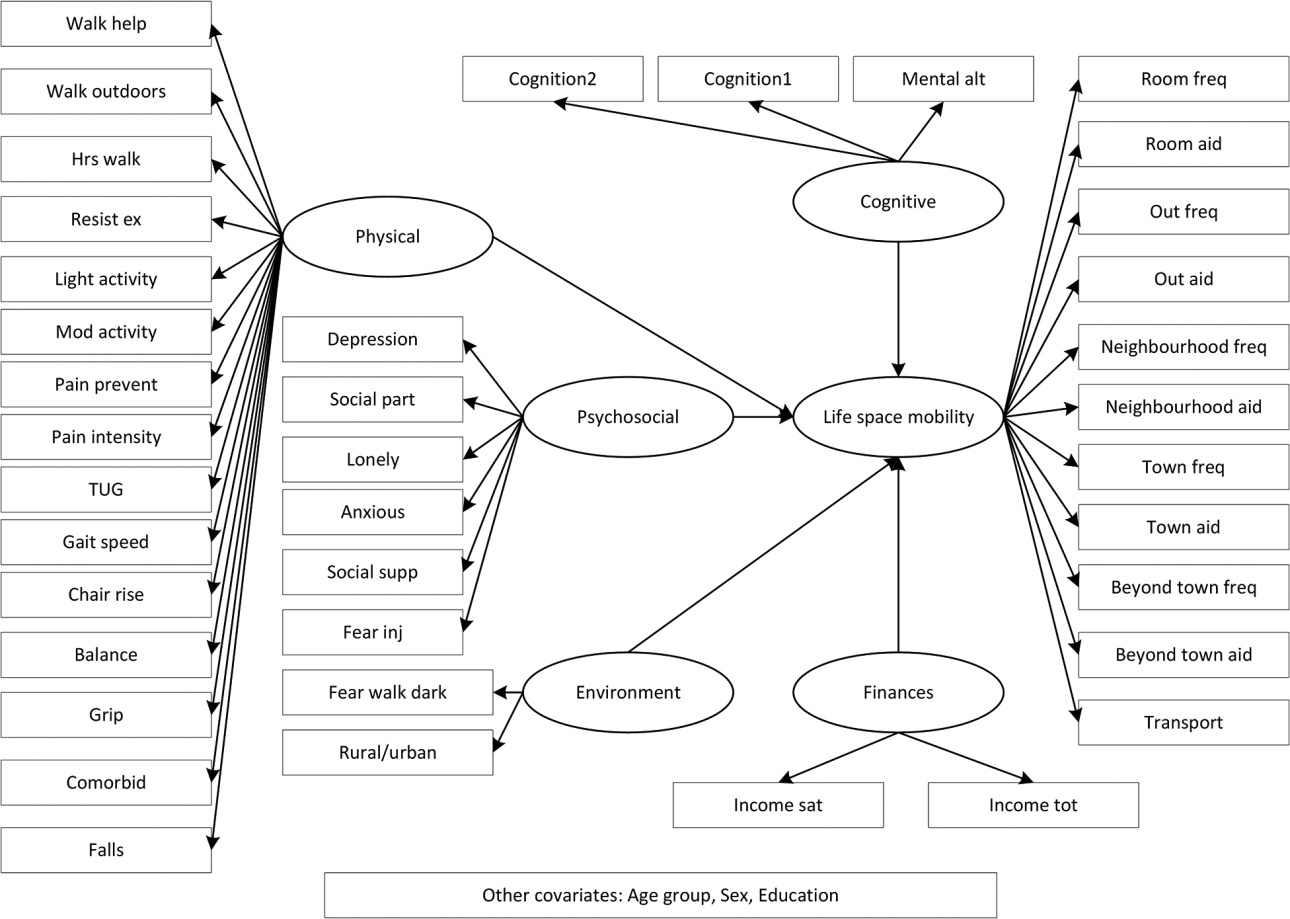
Conceptual model of life space mobility

Items from the CLSA data set representing the five categories of mobility determinants in the theoretical framework (physical, psychosocial, environmental, financial, and cognitive) [12] characterized the same five latent factors in our models. The sixth latent factor (the dependent variable) was life space mobility. Detailed information about CLSA questionnaires and physical assessments is available on the CLSA website [21] and in the cohort profile manuscript [22]. Indicator variables contributing to latent factors are listed in Additional file 1 (Additional Table 1). Our conceptual model of life space mobility is shown in Fig. 1. In this study, individuals who answered yes to “Has a doctor ever told you that you have osteoarthritis in the hip?” and/or “Has a doctor ever told you that you have osteoarthritis in the knee?” in the Comprehensive Site Questionnaire were considered to have OA.

Life Space Index scores were calculated so that 120 represented the highest possible level of life space attainment [23]. For the dependent variable, the latent factor life space mobility was measured by the ten items in the Life Space Index [23] and one measure representing the most common form of transportation used in the past year.

Fifteen measures contributed to the latent factor representing physical capacity. These included self-reported items reflecting frequency and average hours per day spent walking, engaging in resistance exercises, and participating in light and moderate intensity physical activities. In addition, participants provided information about types and numbers of comorbidities and the number of times they had fallen in the past 12 months. They reported on intensity of pain experienced and whether pain influenced participation in activities. Physical assessment measures included Timed Get Up and Go (TUG), gait speed (4 m distance), chair rise test (5 repetitions), single leg stance for balance, and grip strength [21].

Psychosocial influences included the presence of anxiety, frequency of feeling depressed, and frequency of feeling lonely. The availability of social supports and frequency of community-based activity participation were included along with a measure of whether fear of injury contributed to lack of participation.

Environmental factors included rural/urban status and fear of walking alone after dark in their local area. Financial influences were assessed through two questions: total household income, and how well income satisfied basic needs. Cognition was represented by scores on the Rey Auditory Verbal Learning test (immediate and delayed recall of words) [21] and the Mental Alternation Test (consecutive numeric and alphabetical alternations, e.g., A1, B2 etc.) [24].

The mobility framework [12] depicts gender, culture, and biographical influences surrounding all mobility determinants. We included the covariates of age, sex, and level of education in our model to portray cross-cutting factors.

### Statistical analyses

The data were analyzed using SEM in R 3.6.3 for Windows with the *lavaan* package. Ordinal items in the measurement models were recoded so higher values indicated better conditions. Observations of “Don’t know”, “No Answer”, and “Refused” were treated as missing. Eight instances of extreme observations were also treated as missing. Missing data in observed variables in the measurement models were addressed using pairwise deletion and missing data for covariates in the structural models were addressed using listwise deletion. The percent of missing data in all observed variables was less than 10%, except fear of injury preventing participation which was > 30% for both groups. Missing data for covariates in the structural models resulted in about 7% of data being removed from the analysis for the 65 + group and 6% of data removed for the OA group. Before modeling, five continuous variables were rescaled to avoid potential problems caused by large differences of variance.

Descriptive statistics were generated and correlation coefficients for all pairs of variables were calculated to check for collinearity among observed variables. One Life Space Index variable representing the frequency of getting to places outside an individual’s town was not included in the latent factor because it was highly correlated with the variable representing use of aids, equipment, or help from another person to get to places outside one’s town (r = 0.93 for 65 + and r = 0.90 for OA group).

Weighted least square mean and variance estimator with delta parameterization were used to estimate SEM parameters from unweighted CLSA data [25, 26]. Model fit was evaluated using robust chi-square test and comparative fit index (CFI), root mean square error of approximation (RMSEA) and its 90% confidence interval (CI) [27]. The criteria of good overall fit were CFI ≥ 0.9 and RMSEA ≤ 0.08 [27].

First, measurement models were investigated to check if latent factors in the theoretical model were properly constructed using the CLSA variables. Second, a structural model with all (modified) measurement models allowing covariances among the latent factors was fit. Then, the covariates were added to the structural model. Modifications in the models were based on modification index [28] from *lavaan* and knowledge of related literature. To investigate if the latent factors were represented in the same way across males and females (both groups) and across younger and older age groups (OA group), the measurement invariance test (configural, metric, and scalar invariance) was conducted using multi-group analyses.

## Results

### Participant characteristics 65 + group and OA group

Participant characteristics for the 65 + group (n = 11,667) and the OA group (n = 5,560) are presented in Tables 1 and 2, respectively. The 65 + and OA groups overlapped; 57.0% of individuals in the OA group were 65 years of age and older, and 26.5% of people in the 65 + group had OA of the hip or knee.

**Table 1:** Characteristics of 65 + group

Characteristic	Categories/units	*n*	%
Age group	65–74 years	6933	59
	75+	4734	41
Sex	Male	5917	51
	Female	5750	49
Marital Status	Single, never married or never lived with a partner/Windowed/Divorced/Separated	4274	37
	Married/Living with a partner in a common-law relationship	7392	63
Education	Less than secondary school graduation	1007	9
	Secondary school graduation, no post-secondary education	1250	11
	Some post-secondary education	962	8
	Post-secondary degree/diploma	8448	72
Rural/Urban	Urban	10,874	93
	Rural	793	7
General Health	Poor	150	1
	Fair	868	7
	Good	3499	30
	Very good	4800	41
	Excellent	2338	20
Transportation	No driver’s license/Do not drive	1177	11
	Have a driver’s license: Most common transportation: do not drive	1112	10
	Have a driver’s license: Most common transportation: drive a motor vehicle	8557	79
Pain Prevent	NOT free of pain, MOST activities prevented by pain or discomfort	446	4
	NOT free of pain, SOME activities prevented by pain or discomfort	824	7
	NOT free of pain, a FEW activities prevented by pain or discomfort	1360	12
	Free of pain or no activities prevented by pain or discomfort	8968	77
Pain Intensity	Usually severe	485	4
	Moderate	2248	19
	Mild	1764	15
	Usually pain free	7072	61

**Table 2:** Characteristics of OA group

Characteristic	Categories/units	*n*	%
Age group	45–54	661	12
	55–64	1780	32
	65–74	1757	32
	75+	1362	25
Sex	Male	2233	40
	Female	3327	60
Marital Status	Single, never married or never lived with a partner/Windowed/Divorced/Separated	2021	36
	Married/Living with a partner in a common-law relationship	3536	64
Education	Less than secondary school graduation	396	7
	Secondary school graduation, no post-secondary education	526	9
	Some post-secondary education	446	8
	Post-secondary degree/diploma	4192	75
Rural/Urban	Urban	5164	93
	Rural	396	7
General Health	Poor	137	3
	Fair	632	11
	Good	1903	34
	Very good	2104	38
	Excellent	779	14
Transportation	No driver’s license/Do not drive	549	11
	Have a driver’s license: Most common transportation: do not drive	590	11
	Have a driver’s license: Most common transportation: drive a motor vehicle	4109	78
Pain Prevent	NOT free of pain, MOST activities prevented by pain or discomfort	406	7
	NOT free of pain, SOME activities prevented by pain or discomfort	731	13
	NOT free of pain, a FEW activities prevented by pain or discomfort	1052	19
	Free of pain or no activities prevented by pain or discomfort	3333	60
Pain Intensity	Usually severe	382	7
	Moderate	1677	30
	Mild	1135	20
	Usually pain free	2308	42

### Goodness-of-fit of SEM for 65 + group

Items included within latent factors (e.g., the 15 items making up the physical latent factor) were correlated with each other, suggesting the makeup of latent factors was reasonable. See Additional file 2 (Additional Table 2) for details. This was true except for the environmental factor where the two items were not correlated with each other (r = -0.03). Because there were small cells (< 5) in the item for the frequency of getting to other rooms in the home (1–3 times/week and less than once per week), this item was removed along with the item for use of aids to move between rooms.

**Fig. 2:**
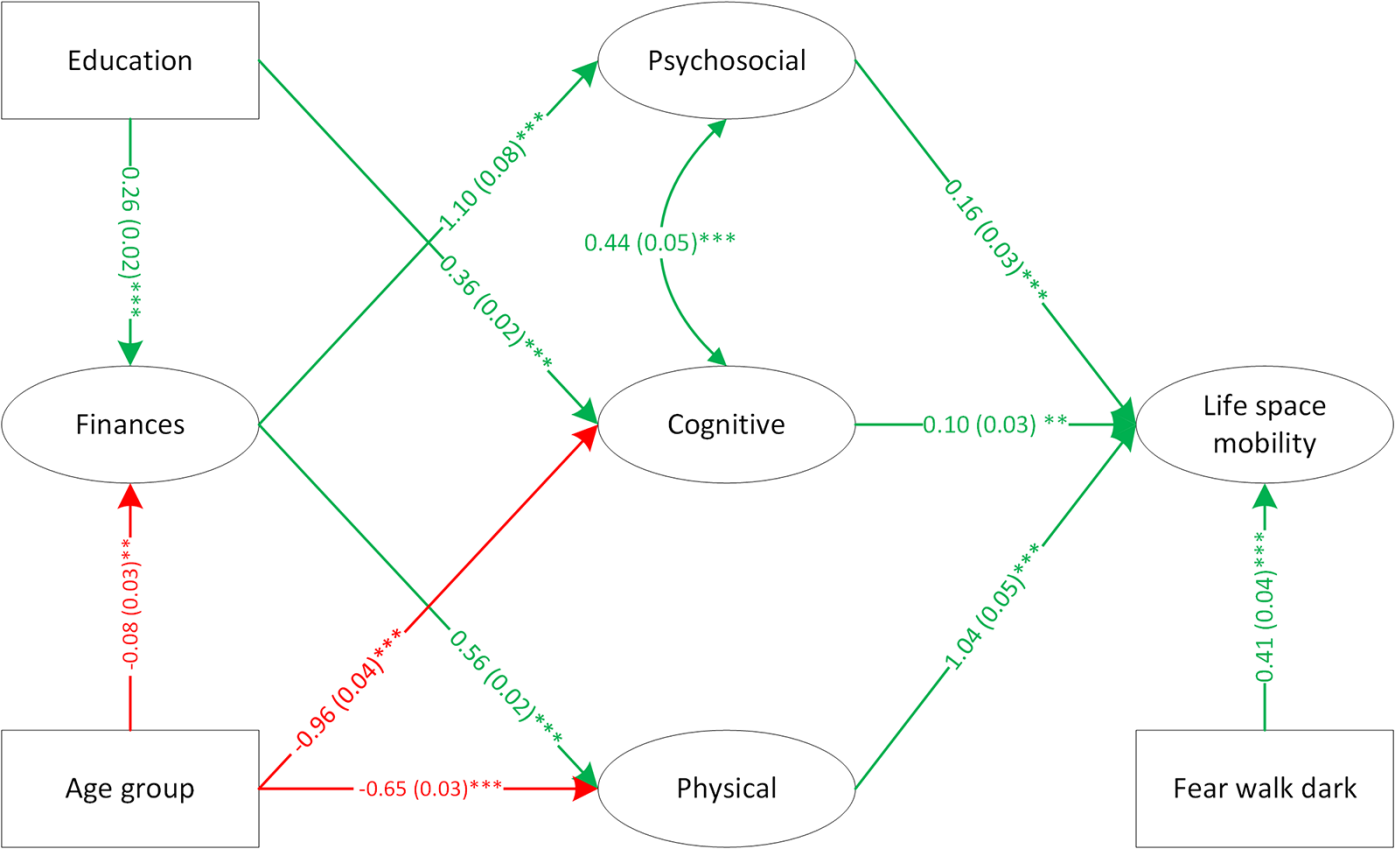
Structural model - final mobility model for 65 + group. *Note*: green indicates positive association; red indicates negative association; cell format: path coefficient (standard error)^significance level^; curved arrow indicates covariance between latent factors; *p < 0.05; **0.01 < p < 0.05: ***p < 0.001. Chi-square (df) = 5591.51 (674), CFI = 0.90, RMSEA (90%) = 0.025 (0.024, 0.026)

The measurement models for life space mobility, physical, psychosocial and cognitive factors had good fit to the data (Additional file 3 – Additional Table 3). These four measurement models, along with the environment latent factor with two indicators and the finances latent factor with two indicators were included in the SEM allowing covariances among latent factors. This model had good fit ((${\chi }^{2}$= 7077.69, df = 634, CFI = 0.96, RMSEA (90% CI) = 0.030 (0.029–0.030)), however, the factor loading on rural/urban from the environmental factor was close to zero (-0.097). So, instead of including environment as a latent factor, the variable associated with feeling afraid to walk alone after dark was treated as a covariate in the structural model. Next, physical, psychosocial, cognitive, finances and fear of walking alone after dark were included in the structural model. The model demonstrated good fit ((${\chi }^{2}$= 5767.38, df = 603, CFI = 0.91, RMSEA (90% CI) = 0.027 (0.026–0.028)) but finances was not significantly related to life space mobility. As documented in previous literature, financial status affects physical and psychosocial health [29]. Therefore, a model that assumed an indirect effect of finances on life space mobility, acting through physical and psychosocial factors, was tested and was found to have good fit ((${\chi }^{2}$ = 6047.24, df = 607, CFI = 0.91, RMSEA (90% CI) = 0.028 (0.027–0.028)). Age group was then added to the structural model as a predictor for the physical, cognitive, and finances factors; and education was added as a predictor for cognition and finances. Age has been shown to influence physical function through its effects on multiple body systems, resulting in decreased strength, flexibility and cardiovascular endurance with increasing age, for example [30]. Age negatively affects cognitive processing speed, reasoning, memory and executive functions, and the presence of common age-related conditions may accelerate cognitive decline [31]. As people retire from the workforce, household income is usually reduced and financial well-being may change depending on demographic factors, whether retirement was planned or unplanned, pensions and personal savings levels [32]. Literature supported adding education as a predictor for cognition [33] and finances [34]. Sex was highly correlated with grip strength (r = 0.87), therefore we did not include sex as a covariate. This final model (Fig. 2) had good fit ((${\chi }^{2}$ = 5591.51, df = 674, CFI = 0.90, RMSEA (90% CI) = 0.025 (0.024–0.026)). Measurement models are in Additional file 4 (Additional Fig. 1).

In the final model, three latent factors (cognitive, psychosocial, physical) and the environmental variable were directly associated with life space mobility; and one latent factor (finances), and two variables (age and education) were indirect influences. People aged 65 years and older with better cognitive, psychosocial, and/or physical health had greater life space mobility. Participants who were less afraid to walk after dark in their local area also demonstrated greater life space mobility. People reporting higher finances had greater psychosocial health and physical health. Higher education was related to better cognitive function and higher finances. Older age was associated with lower financial status, cognition, and physical health. All levels of measurement invariance were retained across sex (Additional file 5 – Additional Table 4), indicating latent factors were measured in the same way in males and females.

### Goodness-of-fit of SEM for OA group

Similar to the model for the 65 + group, items included within latent factor groupings in the OA group were correlated with each other, except for the two items representing environment (r = 0.00, Additional file 6 – Additional Table 5). Because there were empty cells in the item for frequency getting to other rooms in the home, this item was removed along with the item for use of aid to move between rooms. The measurement models for life space mobility, physical, psychosocial and cognitive factors had good fit (Additional file 7 – Additional Table 6). These four measurement models were included in the SEM allowing covariances among latent factors. This model had good fit ((${\chi }^{2}$= 3138.38, df = 443, CFI = 0.96, RMSEA (90% CI) = 0.033 (0.032–0.034)).

**Fig. 3:**
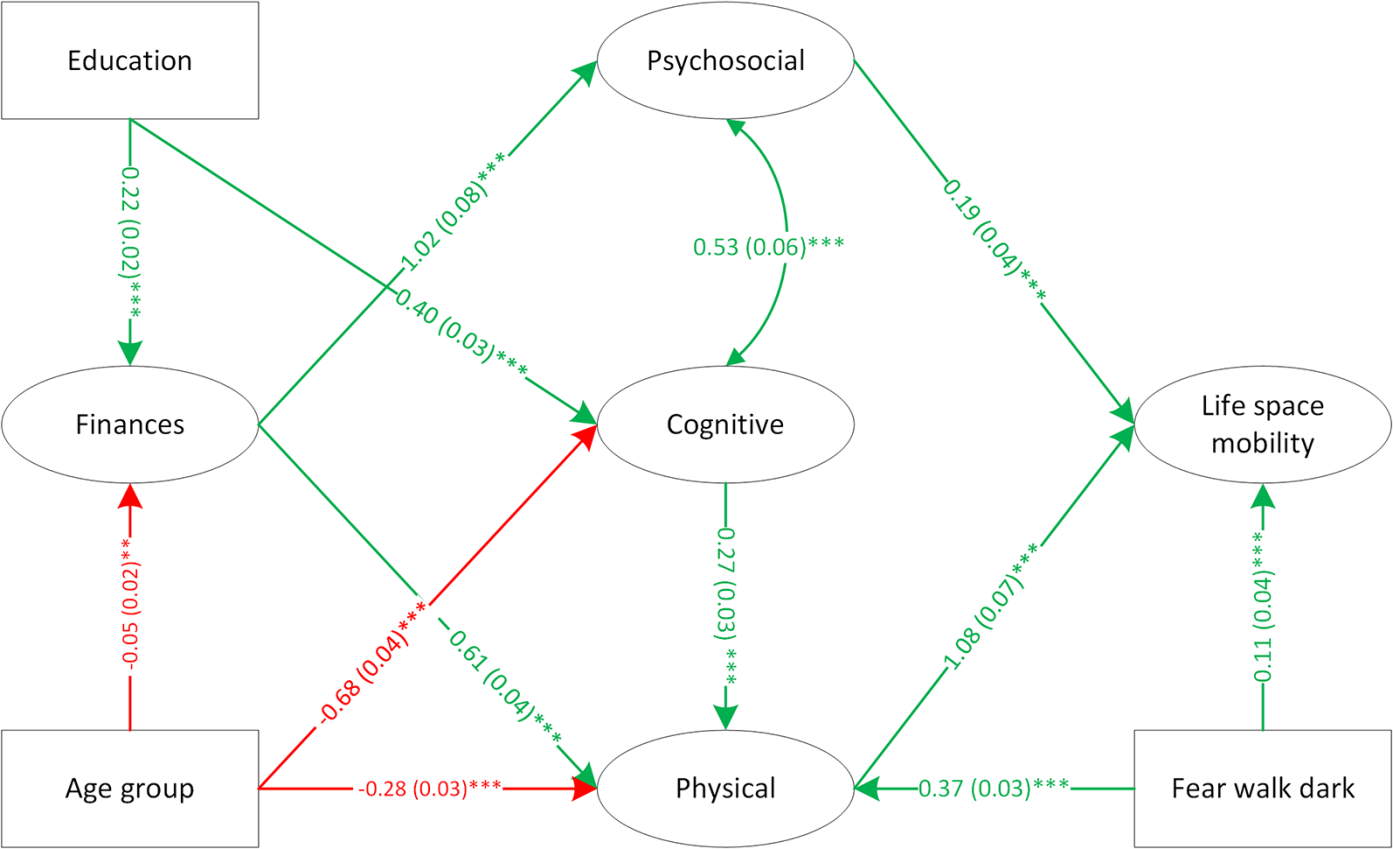
Structural model - final mobility model for OA group. *Note*: green indicates positive association; red indicates negative association; cell format: path coefficient (standard error)^significance level^; curved arrow indicates covariance between latent factors; *p < 0.05; **0.01 < p < 0.05: ***p < 0.001. Chi-square (df) = 3949.13 (601), CFI = 0.90, RMSEA (90%) = 0.032 (0.031, 0.033)

Next, the final model generated for the 65 + group was fit on the data for the OA group. This model did not have adequate fit ((${\chi }^{2}$ = 8549.10, df = 637, CFI = 0.82, RMSEA (90% CI) = 0.047 (0.046–0.048)). Cognition was not significantly related to life space mobility, however it has been shown to be associated with physical capacity [35], therefore, a model that assumed an indirect effect of cognition through physical was fit. This model also did not have adequate fit although goodness-of-fit increased substantially ((${\chi }^{2}$ = 4345.98, df = 602, CFI = 0.88, RMSEA (90% CI) = 0.033 (0.033–0.034)). As suggested by the modification index and with support from the literature, physical health was allowed to regress on the variable representing afraid to walk alone after dark [18, 36]. This final model had good fit ((${\chi }^{2}$=3949.13, df = 601, CFI = 0.90, RMSEA (90% CI) = 0.032 (0.031–0.033)). The final structural model is shown in Fig. 3 and the measurement models are in Additional file 8 – Additional Fig. 2.

In the final model for the OA group, latent factors for psychosocial and physical, and the environmental measure (afraid to walk alone after dark) were directly associated with life space mobility; finances, age, education and cognitive measures were indirectly associated with life space mobility. People with OA who had better psychosocial health and/or physical health had greater life space mobility. Individuals who reported feeling less afraid walking after dark had greater life space mobility and better physical health. Higher cognition was related to better physical health. Participants with stronger financial situations had better psychosocial health and physical health. Higher levels of education were related to higher cognition and higher financial status. Older age was associated with lower financial status, cognition, and physical health. Measurement invariance at all levels was retained across male and female groups and across older and younger age groups (Additional file 9 – Additional Table 7). Therefore, the measurement of the latent factors were the same across sex and age groups.

## Discussion

Models generated with CLSA data verified Webber et al.’s theoretical framework, demonstrating many latent factors that influence life space mobility directly, and others that act indirectly [12]. This represents the second time SEM has been used to evaluate the mobility framework and the first time the outcome of life space mobility has been examined using SEM. Verifying the framework using different sources of data provides additional information about the utility and potential generalizability of the framework. Umstattd Meyer and colleagues modeled personal and community mobility with U.S. Health and Retirement Study data (mean age 74.7 (SD = 7.1)) [16]. They found associations between mobility and personal, physical, psychosocial, environmental, and cognitive factors. Financial status (household income) was not related to personal or community mobility. Umstattd Meyer’s community mobility measure was limited, reflecting only driving habits and availability of a vehicle. CLSA data included many forms of transportation (e.g., driving, arranging rides as a passenger, using public transit, cycling, walking) and many life space destinations (within home, neighborhood, town, outside town) [16]. Participants in the CLSA and in the U.S. Health and Retirement Study also differed significantly in terms of education. More than three-quarters of the United States sample were educated at the level of high school diploma, whereas 80% of CLSA participants had some post-secondary education. These distinctions, along with different variables used to represent latent factors likely explain discrepancies in the resultant models.

Using SEM allowed for inclusion of multiple self-report and observed indicator variables and covariates to be assessed together. Our model for the 65 + group demonstrated direct associations between life space mobility and psychosocial, physical, environmental, and cognitive factors. Variables contributing to psychosocial health in the CLSA data set included measures of depression, anxiety, loneliness, frequency of community activity participation, level of social support and fear of injury influencing participation levels. Literature supports a relationship between psychosocial aspects and mobility. Depression [19, 37], and low levels of social engagement outside the home along with less frequent use of telephone/internet for social purposes [38] are associated with restricted life space. Similarly, lower levels of receiving and giving social support, and smaller social networks and/or less frequent social engagement limit life space mobility [19]. Fear of falling, a psychological factor, was found to be associated with life space mobility in community dwelling older adults in four countries, including Canada [39].

Literature also supports the positive association between physical factors and life space mobility as depicted in our models. For example, Dunlap et al. found significant positive associations between Life Space Index, gait speed, lower extremity power, and 6MWT distances achieved in community-dwelling older adults [13]. Poor balance (difficulty with tandem stand) was associated with lower life space scores. Another study reported low levels of overall physical activity and daily step counts in older adults with restricted independent life space attainment [40].

Similar to Umstattd Meyer et al. (2014), we found that feelings of greater safety in the neighborhood were associated with greater mobility. Other environmental aspects have been shown to be related to reduced life space mobility in cross-sectional studies including the presence of high curbs, dangerous cross-roads, and winter weather [41]. Limited outdoor mobility is also associated with poor sidewalks, heavy traffic, inadequate lighting, lack of benches along routes, and long distances to services [42]. Efforts should be made to continue to collect environmental variables in comprehensive ways to inform future research.

Relationships between cognition and life space mobility are equivocal. Some studies have demonstrated life space restrictions in people with lower cognitive functioning [41, 43], while other findings suggest cognition and life space may not be directly associated; depression, locus of control, gait speed and grip strength can act as intervening mediating or moderating factors [44]. Of note, while the 65 + model demonstrated a direct relationship between cognition and life space mobility, this was not true for the OA model. In the OA model, cognition influenced mobility through associations with psychosocial and physical health. Social frailty, a state of limited social resources and limited social activities or abilities important for meeting social needs [45] is closely tied to executive function and together, social frailty and cognition can influence life space [46]. Positive associations between cognition and physical health in older adults are also well-substantiated [47]. In one meta-analysis of 26 cross-sectional studies including 26,355 participants, measures of physical capacity (e.g., gait speed, TUG) were significantly associated with global cognition (Mini-Mental State Examination or the 3MS modified version) [48].

Our model for the 65 + group demonstrated indirect associations between education, finances, and age group with life space mobility. Previous studies support these relationship findings. Research has demonstrated that higher levels of formal education are positively associated with income [34] and cognitive function throughout adulthood [33]. Literature also supports relationships between finances with psychosocial and physical health. Financial insecurity has been linked to poor mental and physical health (e.g., depression, suicide, psychosis, drug abuse, obesity) [29]. Age has been shown to be negatively associated with physical [30] and cognitive health [31], with household income usually declining as people get older [32].

The theoretical framework suggests that personal factors such as gender, culture, and biographical influences may also impact mobility [12]. Umstattd Meyer et al. found gender was not related to personal and community mobility [16]. Unfortunately, gender was not included in CLSA baseline data acquisition. While the CLSA data did include sex, we were unable to include sex in our models because it was highly correlated with grip strength. However, testing for measurement invariance demonstrated the latent factors were measured in the same way across male and female sex categories in our models. Other personal factors (education, age) and finances were also indirectly associated with life space mobility as supported in previous literature [49, 50].

Structural models for the OA group and the 65 + group were similar. Indeed, there was significant overlap in the samples. Verifying the comprehensive framework in these two samples of older adults provides further information about the utility of the framework. The fact that the models were very similar suggests that life space mobility models for older adults with other chronic conditions may show similar relationships between constructs. We found that cognition was directly related to mobility only in the 65 + group, perhaps reflecting relatively greater influence of cognition on life space attainment in these individuals who were slightly older. The OA model included positive associations between cognitive and physical health (which included pain measures), and the OA group had higher levels of pain intensity and activities prevented by pain (Tables 2; Additional file 3 – Additional Table 3). Research has demonstrated a reciprocal relationship between cognition and chronic pain, such that modifying one’s thinking/attention may regulate pain perception and conversely, chronic pain may interfere with cognitive processes [51]. There was also a positive association between feeling less afraid to walk alone after dark and physical health in the OA model. Individuals with OA affecting the hip and/or knee typically experience limitations in walking [10], which may make them feel more vulnerable and exacerbate fear of walking alone after dark.

Several implications for clinical practice, research, and policy development are suggested by findings from this study. Measures contributing to the physical capacity latent factor included common assessments conducted in rehabilitation and research settings (e.g., TUG, gait speed, chair rise test, grip strength) and questions pertinent to a physical activity history (frequency and time spent walking and engaging in light and moderate intensity activities). Results reinforce the importance for clinicians to measure physical capacity, to focus treatments on improving walking capacity, to take a comprehensive history, and also give direction regarding the types of outcome measures and targeted interventions that should be utilized. Findings encourage clinicians to think beyond the influence of physical factors on mobility. The fact that cognition and psychosocial factors were also directly associated with life space mobility in the 65 + model emphasizes the value of a thorough assessment that takes into consideration depression, anxiety, social supports, extent of community-based participation, and memory abilities. The multitude of factors that influence life space mobility should encourage clinicians and researchers to work in interdisciplinary teams to address needs of older adults. Recognition that finances and education indirectly affect life space mobility is important for researchers (e.g., to understand important demographic information to collect with participants), and for policy makers and community organizations (e.g., to understand broad determinants affecting older adults’ access to communities). The complexity of factors associated with life space mobility beseeches inter-connected societal approaches to improve or maintain mobility in older adults.

Strengths of this study include the large sample of Canadians which allowed for generation of two models (65 + and OA). CLSA data included the life space index questionnaire [23], a commonly used and comprehensive measure. The database also included multiple observed variables to represent most constructs in the theoretical mobility model. Despite this, measures representing financial and environmental determinants were limited. This study utilized data collected in the Comprehensive sample included in the CLSA. While the sample was national in scope, it is not intended to be generalizable to the entire Canadian population because only people living within 25–50 km of the 11 data collection sites (located in 7 provinces) were eligible to participate [52]. This was a relatively highly educated sample, with over 70% in both the 65 + group and the OA group having obtained a post-secondary degree or diploma. The cross-sectional nature of this evaluation precludes making causal inferences, and it should be noted that findings from Canada may also not be generalizable to individuals from other parts of the world.

## Conclusions

We used data from a large population-based sample to verify the highly cited comprehensive framework for mobility [12]. Findings confirm the complex inter-relationship of financial, psychosocial, environmental, physical, cognitive, and personal factors that influence life space mobility. All latent factors representing determinants in the original model were associated with life space mobility when considered together using SEM. Our results support continued use of the framework to conceptualize mobility broadly to foster interdisciplinary research and policy development in diverse contexts including clinical practice, transportation and logistics, built environment design, and community development. Research and clinical practice should avoid unidimensional analyses of factors that influence older adults’ abilities to access their communities. Common outcome measures utilized in the CLSA (e.g., TUG, gait speed, grip strength, Rey Auditory Verbal Learning test, Mental Alternation Test) and questions about depression and anxiety show relevant and significant links to life space mobility. Clinicians and researchers should continue to use these measures and related measures to assess and formulate relevant treatment goals with clients. Programming for older adults should attempt to include opportunities for physical activity, social engagement, and appropriate levels of cognitive challenge. Clinicians, researchers, and policymakers alike should consider the ways in which society can promote physical, emotional, cognitive and financial health for all adults, even in younger age groups, because these factors are instrumental in determining mobility in later years.

### Electronic supplementary material

Below is the link to the electronic supplementary material.

**Supplementary Material 1**: **Additional file 1**: Additional Table 1. Items for initial life space mobility models. **Additional file 2**: Additional Table 2. Correlation coefficient matrix for 65+ group. **Additional file 3**: Additional Table 3. Measurement models for 65+ Group. **Additional file 4**: Additional Fig 1. Measurement models for 65+ group. **Additional file 5**: Additional Table 4. Measurement invariance for sex (males vs. females) for the 65+ group. **Additional file 6**: Additional Table 5. Correlation coefficient matrix for OA group. **Additional file 7**: Additional Table 6. Measurement models for OA Group. **Additional file 8**: Additional Fig 2. Measurement models for OA group. **Additional file 9**: Additional Table 7. Measurement invariance for sex (males vs. females) and age (65− vs. 65+) for the OA group

## Data Availability

The datasets generated and/or analyzed during the current study are available from the Canadian Longitudinal Study on aging (www.clsa-elcv.ca) for researchers who meet the criteria for access to de-identified CLSA data.

## List of abbreviations

CI: Confidence interval
CFI: Comparative fit index
CLSA: Canadian Longitudinal Study on Aging
OA: Osteoarthritis
RMSEA: Root mean square error of approximation
SEM: Structural equation modeling
TUG: Timed Get Up and Go
