# Supplementary material for: Verification of a comprehensive framework for mobility using data from the Canadian Longitudinal Study on Aging: a structural equation modeling analysis
Source: BMC Geriatr. 2023 Dec 8;23:823. doi: 10.1186/s12877-023-04566-x (PMC10704626; doi:10.1186/s12877-023-04566-x)
Supplement: Supplementary file 1 — Supplementary Material 1: Additional file 1: Additional Table 1. Items for initial life space mobility models. Additional file 2: Additional Table 2. Correlation coefficient matrix for 65+ group. Additional file 3: Additional Table 3. Measurement models for 65+ Group. Additional file 4: Additional Fig 1. Measurement models for 65+ group. Additional file 5: Additional Table 4. Measurement invariance for sex (males vs. females) for the 65+ group. Additional file 6: Additional Table 5. Correlation coefficient matrix for OA group. Additional file 7: Additional Table 6. Measurement models for OA Group. Additional file 8: Additional Fig 2. Measurement models for OA group. Additional file 9: Additional Table 7. Measurement invariance for sex (males vs. females) and age (65− vs. 65+) for the OA group [file 12877_2023_4566_MOESM1_ESM.docx]

**Supplementary Information**

**Additional Files**

**Additional file 1**

**Additional Table 1.** Items for initial life space mobility models

| **Latent Factor** | **Label in Models** | **Variable** | **Original Source** | **CLSA Source** | **Description or question wording** |
| --- | --- | --- | --- | --- | --- |
| Physical | Walk help | Ability to walk with or without help | OARS (walking ability) | In-Home  Baseline Questionnaire | Able to walk with help, or able to walk without help.  Help = help of a person or mobility aid. (Combined variables of walking aids required plus walking ability). |
|  | Walk outdoors | Frequency of walking outside home or yard in the past 7 days | PASE | Maintaining Contact Questionnaire | “Over the past 7 days, how often did you take a walk outside your home or yard for any reason? For example, for pleasure or exercise, walking to work, walking the dog, etc.” |
|  | Hrs walk | Average hours per day spent walking | PASE | Maintaining Contact Questionnaire | “On average, how many hours per day did you spend walking?” |
|  | Resist ex | Frequency and average hours per day engaged in resistance exercises | PASE | Maintaining Contact Questionnaire | “Over the past 7 days, how often did you do any exercises specifically to increase muscle strength and endurance, such as lifting weights or push-ups, etc?”  Combined with “On average, how many hours per day did you engage in exercises to increase muscle strength and endurance”? |
|  | Light activity | Frequency and average hours per day engaged in light intensity sports or recreational activities | PASE | Maintaining Contact Questionnaire | “Over the past 7 days, how often did you engage in light sports or recreational activities such as bowling, golf with a cart, shuffleboard, badminton, fishing or other similar activities?” Combined with “On average, how many hours per day did you engage in these light sports or recreational activities?” |
|  | Mod activity | Frequency and average hours per day engaged in moderate intensity sports | PASE | Maintaining Contact Questionnaire | “Over the past 7 days, how often did you engage in moderate sports or recreational activities such as ballroom dancing, hunting, skating, golf without a cart, softball, or other similar activities?”  Combined with “On average, how many hours per day did you engage in these moderate sports or recreational activities?” |
|  | Pain prevent | Degree that presence of pain prevents engagement in activities |  | Maintaining Contact Questionnaire | “How many activities does your pain or discomfort prevent? Would you say none, a few, some, or most?” |
|  | Pain intensity | Intensity of pain experienced |  | Maintaining Contact Questionnaire | “How would you describe the usual intensity of your pain or discomfort? Would you say it is mild, moderate, or severe?”  Combined with “Are you usually free of pain or discomfort?” |
|  | TUG | Total time required to complete Timed Get Up and Go test (in seconds) | Timed Up and Go | Physical Assessment | Total time in seconds required to stand up, walk 3 metres, turn around, return to chair, and sit back down. |
|  | Gait speed | Timed 4 metre walk test (in seconds) | Four metre walk test | Physical Assessment | Time to walk 4 metres expressed as gait speed (metres/second). |
|  | Chair rise | Total time required to complete 5 sit-to-stand movements (in seconds) | Chair rise | Physical Assessment | Total time required to completely stand up and sit down from chair 5 times (in seconds). |
|  | Balance | Single leg standing balance (in seconds) | Standing balance test | Physical Assessment | Best attained time for standing on one leg (in seconds). |
|  | Grip | Grip strength (in kg) | Grip strength | Physical Assessment | Maximum value of three repetitions measured with the dominant hand. |
|  | Comorbid | Number of comorbidities (from list of 39) |  | Comprehensive Site Questionnaire | Number of comorbidities (from list of 39) |
|  | Falls | Number of falls in past 12 months |  | Maintaining Contact Questionnaire | “How many times have you fallen in the past 12 months?” |
| Psychosocial | Depression | Frequency feeling depressed in past week | CES-D 10 | Comprehensive Site Questionnaire | “How often did you feel depressed?” (in the last week). |
|  | Social part | Frequency of community-related activity participation (as a participant) in past 12 months |  | Comprehensive Site Questionnaire | Frequency of participation in any type of community-related activity during the past 12 months (none, daily, weekly, monthly, yearly). |
|  | Lonely | Frequency feeling lonely in past week |  | Comprehensive Site Questionnaire | “How often did you feel lonely” |
|  | Anxious | Agree or disagree with being anxious and easily upset |  | Maintaining Contact Questionnaire | “I see myself as anxious and easily upset” (disagree, agree, neither agree or disagree). |
|  | Social supp | Level of social support | Functional social support – MOS scale | Comprehensive Site Questionnaire | Overall level of functional social support available to the respondent (including all aspects in the MOS Social Support Survey). |
|  | Fear inj | Fear of injury as factor preventing participation in more physical activities |  | Maintaining Contact Questionnaire | “Fear of injury” as answer to “What prevented you from doing physical activities/more physical activities?” |
| Environment | Fear walk dark | Degree of agreement with feeling afraid to walk alone after dark in their local area | HRS | Maintaining Contact Questionnaire | Response to “People would be afraid to walk alone after dark in this area.” |
|  | Rural/urban^a,b^ | Live in a rural area (<10,000 people) or urban area (≥10,000 people) |  | Based on postal code | Five urban classifications combined as ‘urban’ and one rural category. |
| Finances | Income sat | Degree of agreement with income satisfying basic needs |  | Maintaining Contact Questionnaire | “How well do you think that your income currently satisfies your basic needs?” |
|  | Income tot | Estimate of total household income before taxes and deductions in the past 12 months |  | In-Home  Baseline Questionnaire | “What is your best estimate of the total household income received by all household members, from all sources, before taxes and deductions, in the past 12 months?” |
| Cognitive | Cognition1 | Number of words (or variants) correctly recalled (of 15 words provided on a recording) in 90 seconds – Immediate Recall | Rey Auditory Verbal Learning Test (Immediate Recall) | In-Home  Baseline Questionnaire | Number of words (or variants) correctly recalled (of 15 words provided on a recording) in 90 seconds – Immediate Recall |
|  | Cognition2 | Number of words (or variants) correctly recalled (of 15 words previously provided on a recording) in 60 seconds – Delayed Recall | Rey Auditory Verbal Learning Test (Delayed Recall) | In-Home  Baseline Questionnaire | Number of words (or variants) correctly recalled (of 15 words previously provided on a recording) in 60 seconds – Delayed Recall |
|  | Mental alt | Number of correct consecutive numeric and alphabetical alternations in 30 seconds (Mental Alternation Test) | Mental Alternation Test | In-Home  Baseline Questionnaire | Mental Alternation Test (modeled on the Trail making test) - Number of correct consecutive numeric and alphabetical alternations in 30 seconds |
| Life space mobility | Room freq^b^ | Frequency getting to other rooms of home besides the room where you sleep | LSI | In-Home  Baseline Questionnaire | “How often did you get to other rooms of your home  besides the room where you sleep?”  Combined with “During the past four weeks, have you been to other rooms of your home besides the room where you sleep? ” |
|  | Room aid^b^ | Use of aids, equipment, or help from another person to get to other rooms of your home besides the room where you sleep | LSI | In-Home  Baseline Questionnaire | “Did you use aids or equipment, or need help from another person to get to other rooms of your home besides the room where you sleep?”  Combined with “During the past four weeks, have you been to other rooms of your home besides the room where you sleep? ” |
|  | Out freq | Frequency getting to an area outside your home such as your porch, deck or patio, hallway (of an apartment building) or garage | LSI | In-Home  Baseline Questionnaire | “How often did you get to an area outside your home such as your porch, deck or patio, hallway (of an apartment building) or garage, in your own yard or driveway?”  Combined with “During the past four weeks, have you been to an area outside your home such as your porch, deck or patio, hallway (of an apartment building) or garage, in your own yard or driveway? ” |
|  | Out aid | Use of aids, equipment, or help from another person to get to areas outside your home such as your porch, deck or patio, hallway (of an apartment building) or garage | LSI | In-Home  Baseline Questionnaire | “Did you use aids or equipment, or need help from another person to get to an area outside your home such as your porch, deck or patio, hallway (of an apartment building) or garage, in your own yard or driveway?”  Combined with “During the past four weeks, have you been to an area outside your home such as your porch, deck or patio, hallway (of an apartment building) or garage, in your own yard or driveway? |
|  | Neighbourhood freq | Frequency getting to places in neighbourhood | LSI | In-Home  Baseline Questionnaire | “How often did you get to places in your neighbourhood, other than your own yard or apartment building?”  Combined with “During the past four weeks, have you been to places in your neighborhood, other than your own yard or apartment building?” |
|  | Neighbourhood aid | Use of aids, equipment, or help from another person to get to places in neighbourhood |  | In-Home  Baseline Questionnaire | “Did you use aids or equipment, or need help from another person to get to places in your neighbourhood, other than your own yard or apartment building?”  Combined with “During the past four weeks, have you been to places in your neighborhood, other than your own yard or apartment building?” |
|  | Town freq | Frequency getting to places outside neighbourhood but within town | LSI | In-Home  Baseline Questionnaire | “How often did you get to places outside your neighbourhood, but within your town?”  Combined with “During the past four weeks, have you been to places outside your neighborhood, but within your town?” |
|  | Town aid | Use of aids, equipment, or help from another person to get to places outside neighbourhood but within town | LSI | In-Home  Baseline Questionnaire | “Did you use aids or equipment, or need help from another person to get to places outside your neighbourhood, but within your town?”  Combined with “During the past four weeks, have you been to places outside your neighborhood, but within your town?” |
|  | Beyond town freq^c^ | Use of aids, equipment, or help from another person to get to places outside your town | LSI | In-Home  Baseline Questionnaire | “How often did you get to places outside your town?”  Combined with “During the past four weeks, have you been to places outside your town?” |
|  | Beyond town aid | Use of aids, equipment, or help from another person to get to places outside your town | LSI | In-Home  Baseline Questionnaire | “Did you use aids or equipment, or need help from another person to get to places outside your town?”  Combined with “During the past four weeks, have you been to places outside your town?” |
|  | Transport | Most common form of transportation in past year | Older and Wiser Driver Questionnaire | Maintaining Contact Questionnaire | “In the past year, what was your most common form of transportation?” (driving a vehicle, passenger in a vehicle, public transit, taxi, cycling, walking) |
| Age Group ^d^ | Age | Age |  | In-Home  Baseline Questionnaire | “What is your age?”  Age classified in groups (45-54, 55-64, 65-74, 75-85 years of age). |
| Sex^d^ | Sex | Sex |  | In-Home  Baseline Questionnaire | “Are you male or female?” |
| Level of Education ^d^ | Education | Education |  | In-Home  Baseline Questionnaire | “What is the highest degree, certificate, or diploma you have obtained?” |

*Note:* ^a^ variable not in final 65+ model; ^b^ variable not in final OA model; ^c^ item not included in Life space mobility latent factor (highly correlated with Beyond town aid); ^d^ covariate added to structural model; LSI = Life Space Index; OARS= Older Americans Resources and Services; PASE = Physical Activity Scale for the Elderly; CES-D 10 = Center for Epidemiologic Studies Short Depression Scale; HRS = Health and Retirement Survey; MOS = Medical Outcomes Study. Older and Wiser Driver Questionnaire is part of the BASeline Survey of Seniors developed by the Centre on Aging – University of Victoria.

**Additional file 2**

**Additional Table 2.** Correlation coefficient matrix for 65+ group (double click on Table to enable functionality)

**Additional file 3**

**Additional Table 3**. Measurement models for 65+ Group

| Measurement Model | $\boldsymbol{\chi}^{\boldsymbol{2}}$ | df | CFI | RMSEA (90% CI) |
| --- | --- | --- | --- | --- |
| Life space mobility | 383.22 | 30 | 0.99 | 0.032 (0.029 – 0.035) |
| Physical | 1006.65 | 84 | 0.99 | 0.031 (0.029 – 0.032) |
| Psychosocial | 5.41 | 5 | 1 | 0.003 (0.000 – 0.013) |
| Cognitive | 7.988 | 1 | 1 | 0.025 (0.011 – 0.042) |

*Note:* $\chi^{2}$= chi-square, df = degrees of freedom, CFI = comparative fit index, RMSEA = root mean square error of approximation, CI = confidence interval

**Additional file 4**

**Additional Figure 1.** Measurement models for 65+ group

**
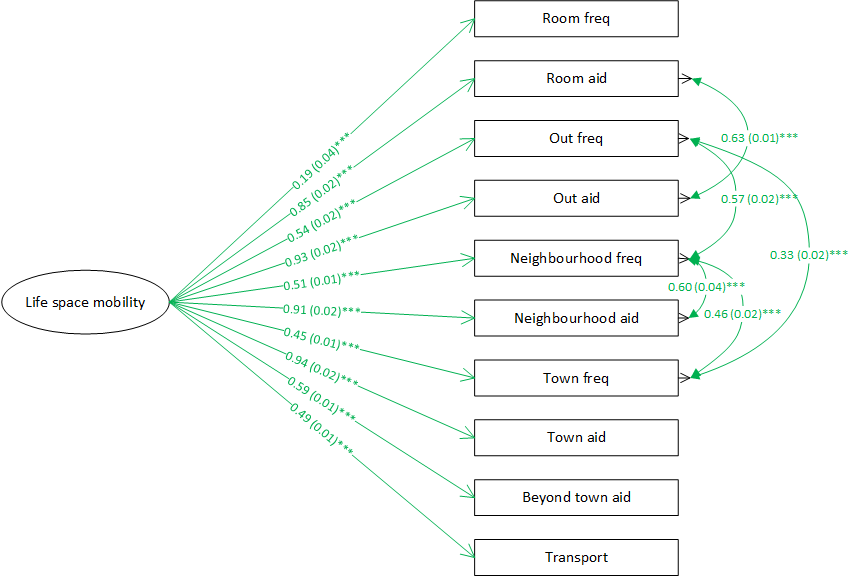
**

**
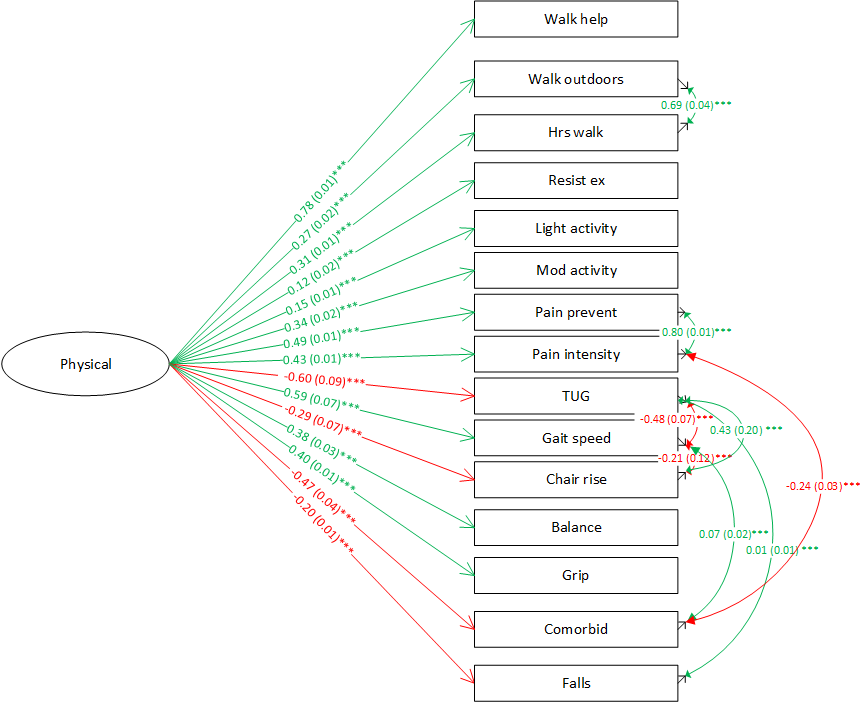

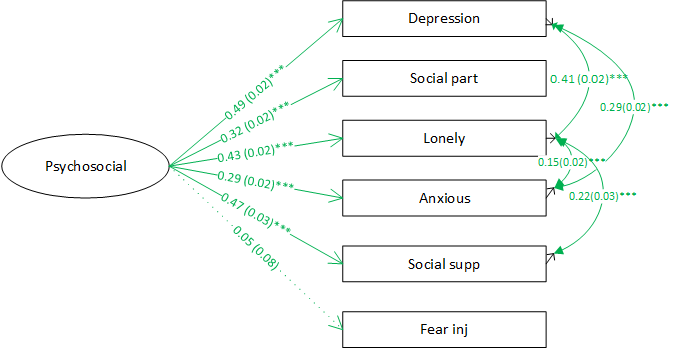
**

**
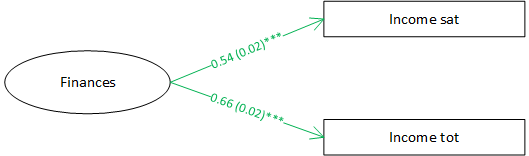
**

*Note*: green indicates positive association; red indicates negative association; cell format: standardized factor loading (standard error)­­^significance level^; curved arrows indicates error covariances; * *p* <.05, ** .01 < *p* < .05, *** *p* < .001

**Additional file 5**

**Additional Table 4.** Measurement invariance for sex (males vs. females) for the 65+ group

| Invariance Type | Robust chisq (df) | ∆ Robust chisq (∆df) | | RMSEA | CFI | ∆ CFI |
| --- | --- | --- | --- | --- | --- | --- |
| Configural | 5589.00 (1348) |  | 0.023 | | 0.91 |  |
| Metric | 5096.65 (1379) | -492.35 (31) | 0.021 | | 0.92 | ≥-0.01 |
| Scalar | 5358.33 (1450) | -261.68 (71) | 0.021 | | 0.92 | ≥-0.01 |

*Note*: chisq = chi square, df = degrees of freedom, ∆ robust chisq = change in robust chi square, ∆df = change in degrees of freedom, RMSEA = root mean square error of approximation, ∆ CFI = change in comparative fit index.

**Additional file 6**

**Additional Table 5.** Correlation coefficient matrix for OA group (double click on Table to enable functionality)

**Additional file 7**

**Additional Table 6.** Measurement models for OA Group

| Measurement Model | $\boldsymbol{\chi}^{\boldsymbol{2}}$ | df | CFI | RMSEA (90% CI) |
| --- | --- | --- | --- | --- |
| Life space mobility | 95.09 | 16 | 1 | 0.030 (0.024 – 0.036) |
| Physical | 680.44 | 84 | 0.98 | 0.036 (0.033 – 0.038) |
| Psychosocial | 14.12 | 5 | 1 | 0.003 (0.000 – 0.013) |
| Cognitive | 0.59 | 1 | 1 | 0.000 (0.000 – 0.033) |

*Note:* $\chi^{2}$= chi-square, df = degrees of freedom, CFI = comparative fit index, RMSEA = root mean square error of approximation, CI = confidence interval

**Additional file 8**

**Additional Figure 2.** Measurement models for OA group


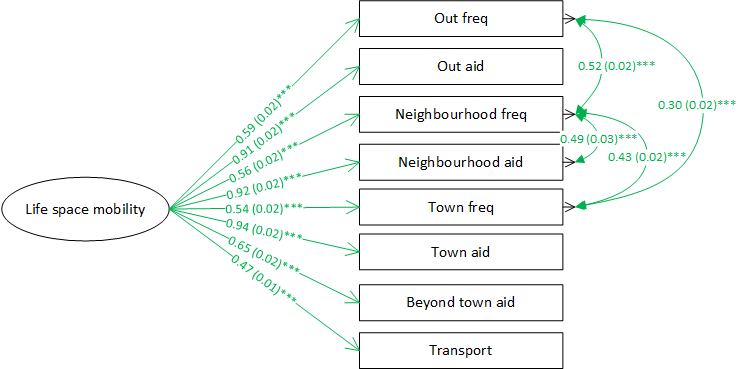


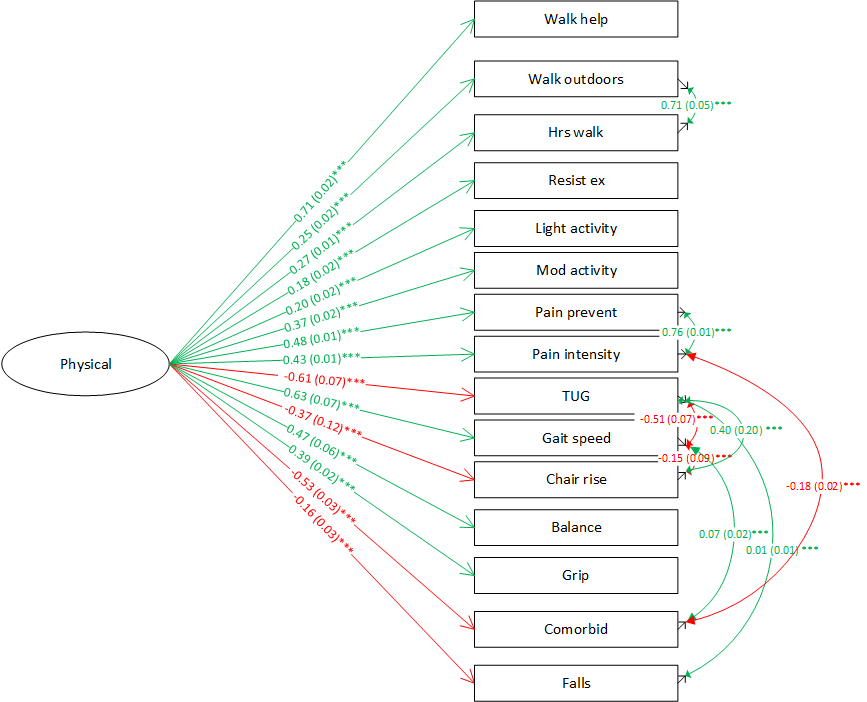

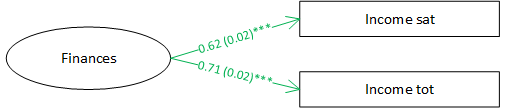


*Notes.* Green indicates positive association; red indicates negative association; cell format: standardized factor loading (standard error)­­^significance level^; curved arrows indicates error covariances; * *p* <.05, ** .01 < *p* < .05, *** *p* < .001

**Additional file 9**

**Additional Table 7.** Measurement invariance for sex (males vs. females) and age (65- vs. 65+) for the OA group

| Invariance Type | Robust chisq (df) | ∆ Robust chisq (∆df) | | RMSEA | CFI | ∆ CFI |
| --- | --- | --- | --- | --- | --- | --- |
| Sex - Configural | 3962.70 (1202) | |  | 0.029 | 0.91 |  |
| Sex - Metric | 3640.35 (1231) | | -322.35 (29) | 0.027 | 0.92 | ≥ -0.01 |
| Sex - Scalar | 3801.85 (1296) | | 161.50 (65) | 0.026 | 0.91 | ≥ -0.01 |
| Age - Configural | 2865.60 (1202) | |  | 0.022 | 0.91 |  |
| Age - Metric | 2647.06 (1231) | | -218.54 (29) | 0.020 | 0.92 | ≥ -0.01 |
| Age - Scalar | 2751.77 (1296) | | 104.71 (65) | 0.020 | 0.92 | ≥ -0.01 |

*Note*: chisq = chi square, df = degrees of freedom, ∆ robust chisq = change in robust chi square, ∆df = change in degrees of freedom, RMSEA = root mean square error of approximation, ∆ CFI = change in comparative fit index.
